# Choroidal Thickening and Reduced Macular Blood Flow in Children with Hyperopic Anisometropic Amblyopia

**DOI:** 10.3390/jcm15052085

**Published:** 2026-03-09

**Authors:** Ryuya Hashimoto, Juri Kawamura, Naoki Fujioka, Kazufumi Tanaka, Moe Nunose, Sara Imai, Serika Moriyama, Ryo Yamazaki, Asato Hirota, Fumihiko Yagi

**Affiliations:** Department of Ophthalmology, Toho University Sakura Medical Center, Chiba 285-8741, Japan

**Keywords:** ophthalmic imaging, anisometropic amblyopia, hyperopia, choroidal blood flow, laser speckle flowgraphy

## Abstract

**Background/Objectives:** This study aimed to evaluate macular choroidal blood flow dynamics and structural alterations in children with hyperopic anisometropic amblyopia and compare these findings with those of the fellow eyes. **Methods:** This retrospective observational study included 36 eyes from 18 children (mean age: 4.9 years) with unilateral hyperopic anisometropic amblyopia. Central choroidal thickness (CCT) was measured using enhanced depth imaging optical coherence tomography. Macular choroidal hemodynamics were assessed using laser speckle flowgraphy. Mean blur rate (MBR) was used as an index of blood flow, whereas beat strength (BS) was used as a measure of pulsatility. Ocular perfusion pressure (OPP) was also calculated. All parameters were compared between amblyopic and fellow eyes. **Results:** Amblyopic eyes demonstrated significantly greater CCT compared with fellow eyes (407.6 ± 84.9 µm vs. 326.4 ± 79.1 µm). Conversely, macular MBR was significantly lower in amblyopic eyes (9.28 ± 3.60 AU vs. 10.94 ± 4.68 AU), as was BS (5.73 ± 3.07 AU vs. 7.28 ± 3.59 AU). No significant differences were observed in central retinal thickness or OPP between amblyopic and fellow eyes. In amblyopic eyes, CCT was not significantly correlated with macular MBR or BS. **Conclusions:** Amblyopic eyes exhibited significant central choroidal thickening accompanied by reduced macular blood flow and pulsatility. These findings suggest that localized macular hemodynamic dysregulation may contribute to the pathophysiology of hyperopic anisometropic amblyopia.

## 1. Introduction

Anisometropia, defined as a significant interocular difference in refractive error, represents a major risk factor for unilateral amblyopia in children [1]. During the critical period of visual development, refractive imbalance results in a persistent disparity in retinal image quality between the two eyes, leading to selective cortical suppression and impaired visual maturation [1]. Pediatric amblyopia affects approximately 1–5% of children worldwide [2] and remains a significant public health concern, as delayed diagnosis and intervention are associated with markedly reduced treatment efficacy. Among the various clinical subtypes, hyperopic anisometropic amblyopia warrants particular attention, as asymmetric axial growth and refractive development predispose the affected eyes to permanent visual deficits and binocular dysfunction. Consequently, elucidating the underlying pathophysiology of hyperopic anisometropic amblyopia is of considerable clinical and scientific importance.

With regard to the pathophysiology of hyperopic anisometropic amblyopia, structural alterations of the choroid—particularly increased central choroidal thickness (CCT)—have been consistently reported. Spectral-domain optical coherence tomography (SD-OCT) studies have demonstrated that CCT is significantly greater in amblyopic eyes than in fellow or emmetropic eyes [3], a finding that has also been confirmed in Japanese pediatric populations [4,5]. Furthermore, investigations of choroidal vascular structure have demonstrated a relative enlargement of the vascular luminal area in amblyopic eyes, suggesting the presence of condition-specific histological changes [6]. However, the association between these morphological alterations and macular choroidal perfusion has not been clarified, and the hemodynamic characteristics of amblyopic eyes remain poorly understood.

Laser speckle flowgraphy (LSFG) is a noninvasive imaging modality that quantifies retinal and choroidal blood flow by calculating the mean blur rate (MBR), derived from temporal fluctuations in laser speckle patterns on the fundus [7,8]. Additionally, extraction of the beat strength (BS)—the pulsatile component of the MBR waveform—allows evaluation of choroidal blood flow pulsatility [9]. BS is regarded as a physiological index reflecting the resistive properties and vascular compliance of the choroidal microvasculature [9]. LSFG has been widely applied in clinical research to detect subtle alterations in choroidal circulation across a range of chorioretinal disorders [10,11,12,13,14]. More recently, the reproducibility of LSFG measurements in pediatric populations has been established [15], supporting its applicability for evaluating choroidal perfusion in school-aged children. Despite consistent evidence of subfoveal choroidal thickening in hyperopic anisometropic amblyopia, no previous studies have directly and quantitatively evaluated macular choroidal blood flow in this condition. Consequently, only a few studies have concurrently evaluated both structural and hemodynamic choroidal parameters within the same cohort, highlighting a critical gap in current evidence.

This study aimed to elucidate the relationship between choroidal structure and hemodynamic dynamics in children with treatment-naïve hyperopic anisometropic amblyopia. Macular choroidal blood flow was quantitatively assessed using laser speckle flowgraphy, in conjunction with measurements of CCT and retinal thickness obtained via enhanced depth imaging OCT (EDI-OCT) and calculations of ocular perfusion pressure (OPP). A comprehensive comparative analysis of choroidal structural and hemodynamic parameters was subsequently performed between amblyopic and fellow eyes.

## 2. Materials and Methods

### 2.1. Study Design and Participants

This retrospective, observational study was conducted at the Department of Ophthalmology, Toho University Sakura Medical Center, between May 2016 and March 2024; our cohort was strictly limited to completely treatment-naïve children. A total of 18 children (36 eyes), comprising 18 amblyopic eyes and 18 fellow eyes, diagnosed with unilateral hyperopic anisometropic amblyopia were enrolled in this study. Amblyopia was defined as best-corrected visual acuity (BCVA) below the age-appropriate normative threshold in the absence of any organic ocular abnormalities, in accordance with previously established criteria [4,5]. Anisometropia was defined as an interocular difference in spherical equivalent of ≥1.50 diopters (D), excluding differences attributable to astigmatism. In all patients, the BCVA of the amblyopic eye was 20/30 or worse or at least 2 lines worse than the fellow eye.

Cycloplegic refraction was performed in all patients using either 0.5% or 1% atropine sulfate (instilled twice daily for 7 days) or 1% cyclopentolate hydrochloride (instilled three times on the day of examination) to ensure complete cycloplegia. Refractive values, expressed as spherical equivalent under cycloplegic conditions, were used for all statistical analyses.

Ocular alignment was assessed using cover and prism cover tests, and patients with manifest strabismus were strictly excluded. Comprehensive ophthalmic examinations—including visual acuity assessment, refraction, axial length measurement, retinal and choroidal structural imaging, and macular choroidal blood flow measurements—were performed on the same day. Eyes with insufficient image quality for reliable analysis were excluded. Additional exclusion criteria included strabismic or deprivation amblyopia, myopia, congenital ocular anomalies, retinopathy of prematurity, and any systemic condition known to potentially affecting ocular circulation.

### 2.2. Ethical Considerations

The study was approved by the Ethics Committee of Toho University Sakura Medical Center (approval no. S24060; 5 November 2024) and conducted in accordance with the tenets of the Declaration of Helsinki. The study details were publicly disclosed on the institutional website, providing eligible patients and their guardians with the opportunity to opt out of the study.

### 2.3. Ophthalmic Examinations

All ophthalmic examinations were performed on the same day under pharmacologically induced mydriasis. Visual acuity was assessed using a standard Landolt C chart and converted to the logarithm of the minimum angle of resolution (logMAR) for statistical analysis. Refractive error was measured with an autorefractometer and expressed as the spherical equivalent. Intraocular pressure (IOP) was determined using a non-contact tonometer (NCT-200; Nidek, Gamagori, Japan), and axial length was measured using an optical biometer (OA-2000; Tomey, Nogoya, Japan).

OPP was calculated using mean arterial pressure (MAP), which was derived from systolic blood pressure (SBP) and diastolic blood pressure (DBP) measured in a resting seated position, according to the following formula: MAP = DBP + 1/3 (SBP − DBP). Subsequently, OPP was determined using the formula: OPP = 2/3 MAP − IOP [16].

### 2.4. Retinal and Choroidal Imaging

**Figure 1:**
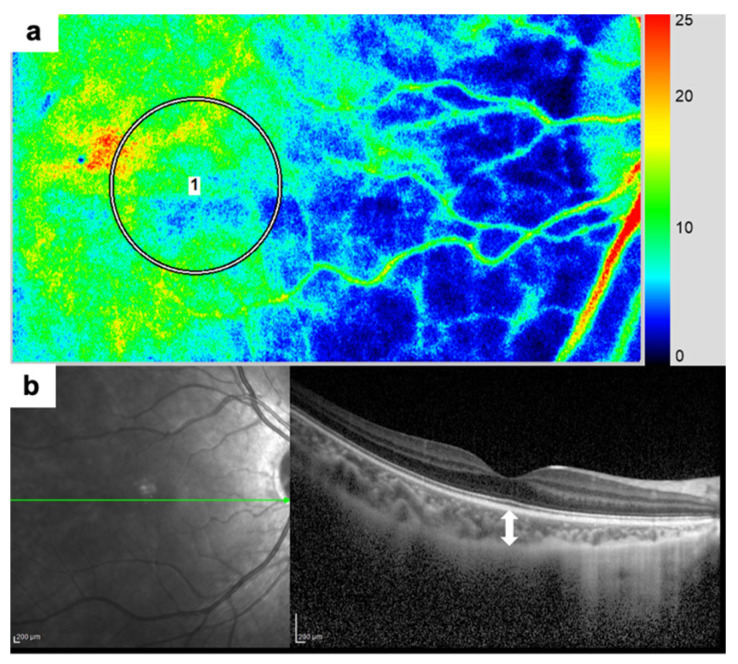
Measurement of choroidal parameters using laser speckle flowgraphy (LSFG) and enhanced depth imaging optical coherence tomography (EDI-OCT). (**a**) LSFG composite map. A circular region of interest was centered on the fovea to quantify macular choroidal hemodynamics. The mean blur rate and beat strength were calculated within this ROI. (**b**) Horizontal EDI-OCT B-scan centered on the fovea. Central choroidal thickness was measured manually as the perpendicular distance from the outer border of the retinal pigment epithelium to the chorioscleral interface (white arrow). Abbreviations: LSFG, laser speckle flowgraphy; EDI-OCT, enhanced depth imaging optical coherence tomography; ROI, region of interest; MBR, mean blur rate; CCT, central choroidal thickness.

Structural assessment of the retina and choroid was conducted using SD-OCT with enhanced depth imaging (EDI-OCT; Spectralis HRA + OCT, Heidelberg Engineering, Heidelberg, Germany). Horizontal B-scans centered on the fovea were acquired to measure central retinal thickness (CRT) and CCT (Figure 1). CCT was defined as the perpendicular distance from the outer border of the retinal pigment epithelium to the chorioscleral interface at the fovea. Meanwhile, CRT was measured from the internal limiting membrane to the outer border of the retinal pigment epithelium at the same location. All measurements were manually performed by two independents (J.K. and A.H.), masked examiners, and the mean values were used for analysis.

### 2.5. Choroidal Blood Flow Imaging

Macular choroidal hemodynamics were evaluated using LSFG-NAVI (Softcare Co., Ltd., Fukuoka, Japan). LSFG is a noninvasive technique that quantifies blood flow velocity based on the temporal variations in laser speckle patterns generated by erythrocyte movement. The MBR, an index of blood flow [8], and BS, reflecting pulsatile blood flow derived from the MBR waveform synchronized with the cardiac cycle, were evaluated [9].

To minimize diurnal variations in choroidal hemodynamics, all measurements were conducted between 13:00 and 16:00. A circular region of interest was centered on the fovea, and data were analyzed using LSFG Analyzer software (version 3.5.0.0).

### 2.6. Statistical Analysis

All statistical analyses were performed using GraphPad Prism (version 10, GraphPad Software, Boston, MA, USA). Data distribution was assessed for normality using the Shapiro–Wilk test. Differences between amblyopic and fellow eyes were evaluated using the paired *t*-test for normally distributed variables and the Wilcoxon signed-rank test for non-normally distributed variables. Associations between CCT and blood flow parameters (macular MBR and BS) were assessed using Pearson’s correlation coefficient. A two-tailed, *p*-value of <0.05 was considered significant.

### 2.7. Use of Generative AI in the Writing Process

During the preparation of this manuscript, the authors used Gemini 1.5 Pro (Google) for the purpose of proofreading and refining the structure of the document. The authors have reviewed and edited the output as needed and take full responsibility for the content of this publication.

## 3. Results

### 3.1. Participant Characteristics

A total of 18 patients (10 girls and 8 boys) were included in this study (Table 1). The participants’ ages ranged from 2 to 10 years, with a mean age of 4.94 ± 2.07 years. The mean BCVA (logMAR) values were 0.42 ± 0.31 in the amblyopic eyes and 0.018 ± 0.18 in the fellow eyes. The spherical equivalent refractive error values were +6.49 ± 1.26 D in the amblyopic eyes and +2.82 ± 2.30 D in the fellow eyes, with the amblyopic eyes being significantly more hyperopic (*p* < 0.0001). Axial length measurements were 20.74 ± 0.66 mm in the amblyopic eyes and 22.12 ± 1.15 mm in the fellow eyes, with the fellow eyes being significantly longer (*p* < 0.0001). No significant differences were observed between amblyopic and fellow eyes in terms of IOP (IOP: 14.7 ± 3.0 vs. 15.3 ± 3.0 mmHg, *p* = 0.229) or OPP (OPP: 35.4 ± 11.9 vs. 34.8 ± 11.5 mmHg, *p* = 0.245).

### 3.2. Retinal and Choroidal Structure

**Figure 2:**
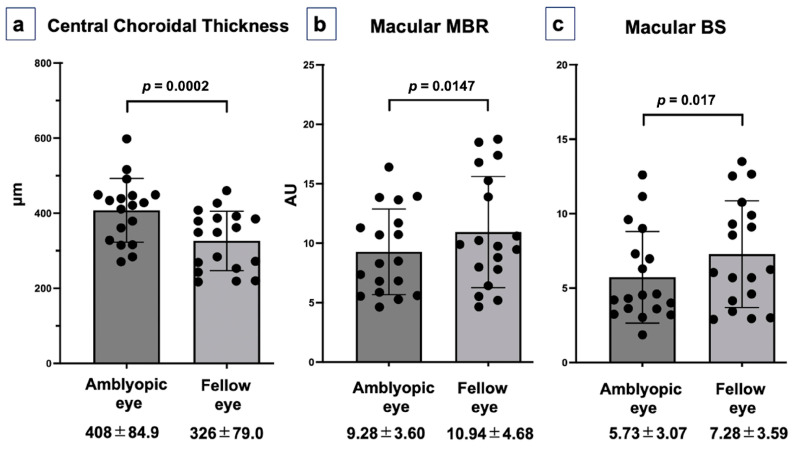
Comparison of choroidal structural and hemodynamic parameters between amblyopic and fellow eyes. (**a**) Central choroidal thickness. The amblyopic eyes demonstrated significantly greater CCT compared with the fellow eyes (*p* = 0.0002). (**b**) Macular mean blur rate (MBR). Amblyopic eyes exhibited significantly lower macular MBR compared with the fellow eyes (*p* = 0.0147). (**c**) Macular beat strength. The pulsatile component (BS) was likewise significantly reduced in the amblyopic eyes (*p* = 0.017). Data are expressed as the mean ± standard deviation, with individual data points overlaid to illustrate the distribution. Abbreviations: CCT, central choroidal thickness; MBR, mean blur rate; BS, beat strength; AU, arbitrary unit.

The CRT values were 207.9 ± 19.8 µm in the amblyopic eyes and 203.1 ± 15.3 µm in the fellow eyes, with no significant difference observed (*p* = 0.133) (Table 1). By contrast, the CCT values were 407.6 ± 84.9 µm in the amblyopic eyes and 326.4 ± 79.1 µm in the fellow eyes, indicating significantly greater CT in the amblyopic eyes (*p* = 0.0002) (Figure 2).

### 3.3. Choroidal Blood Flow and Pulsatility

The macular MBR values were 9.28 ± 3.60 AU in the amblyopic eyes and 10.9 ± 4.68 AU in the fellow eyes, with significantly higher values observed in fellow eyes (*p* = 0.0147) (Figure 2). Similarly, the macular BS values were 5.73 ± 3.07 in the amblyopic eyes and 7.28 ± 3.59 in the fellow eyes, with fellow eyes also showing significantly higher values (*p* = 0.017).

### 3.4. Correlations Between Choroidal Blood Flow and Thickness

The relationship between CCT and macular choroidal hemodynamic parameters was evaluated in the amblyopic eyes. No significant correlation was observed between CCT and macular MBR (*p* = 0.565) or macular BS (*p* = 0.698).

### 3.5. Representative Patient

**Figure 3:**
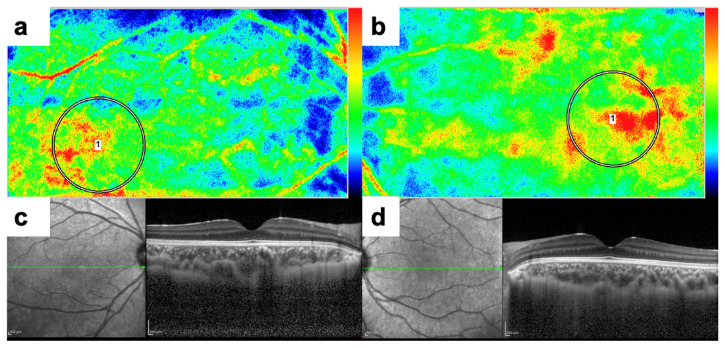
Representative images of a 6-year-old boy with hyperopic anisometropic amblyopia. (**a**) LSFG color map of the amblyopic eye (right). The macular region shows reduced signal intensity (cooler colors), indicating decreased blood flow (MBR = 13.65 AU). (**b**) LSFG color map of the fellow eye (left). The macular region exhibits higher signal intensity (warmer colors), reflecting greater blood flow (MBR = 16.80 AU). (**c**) EDI-OCT B-scan of the amblyopic eye (right). Marked choroidal thickening is observed (CCT = 447 µm). (**d**) EDI-OCT B-scan of the fellow eye (left). The choroid is comparatively thinner (CCT = 349 µm). Abbreviations: LSFG, laser speckle flowgraphy; EDI-OCT, enhanced depth imaging optical coherence tomography; MBR, mean blur rate; CCT, central choroidal thickness; AU, arbitrary unit.

Figure 3 illustrates representative LSFG and EDI-OCT findings from a 6-year-old boy with hyperopic anisometropic amblyopia. The amblyopic eye (right) exhibited a decimal BCVA of 0.6, a spherical equivalent of +4.25 D, and an axial length of 20.88 mm. By contrast, the fellow eye (left) had a decimal BCVA of 1.2, a spherical equivalent of +2.25 D, and an axial length of 23.26 mm. The OPP was comparable between the eyes (31.6 mmHg in the amblyopic eye vs. 34.7 mmHg in the fellow eye). Structurally, CRT was comparable between the eyes (194 µm in the amblyopic eye vs. 209 µm in the fellow eye), whereas CCT was markedly greater in the amblyopic eye (447 µm) compared with the fellow eye (349 µm). Despite this structural thickening, macular hemodynamic parameters were reduced in the amblyopic eye, with an MBR of 13.65 AU vs. 16.80 AU in the fellow eye and a BS of 7.30 AU vs. 9.10 AU in the fellow eye.

## 4. Discussion

This study evaluated macular choroidal hemodynamics using LSFG, whereas choroidal structure was assessed using EDI-OCT in 36 eyes of 18 children with treatment-naïve hyperopic anisometropic amblyopia. Morphological and hemodynamic characteristics were compared between amblyopic and fellow eyes. Amblyopic eyes exhibited significantly greater CCT compared with the fellow eyes, whereas macular blood flow and the pulsatile component were significantly reduced. Conversely, no significant interocular differences were observed in OPP. Furthermore, the relationship between CCT and macular choroidal hemodynamic parameters was further analyzed in amblyopic eyes, revealing no significant correlation with either macular MBR or BS. These findings suggest that choroidal thickening observed in amblyopic eyes occurs independently of macular choroidal blood flow and pulsatility. This indicates that structural changes in the choroid cannot be fully explained by hemodynamic dynamics alone.

The increased CCT observed in pediatric hyperopic anisometropic amblyopia in this study aligns with the findings of previous studies. Several studies using SD-OCT have demonstrated that the choroid is significantly thicker in amblyopic eyes compared with fellow or age-matched normal eyes. This difference persisted even after adjusting for axial length [3,4,5]. Furthermore, analyses of choroidal vascular structure have suggested a relative expansion of the vascular luminal area in amblyopic eyes, implicating tissue remodeling as a structural change specific to amblyopia [6]. In the present study, amblyopic eyes exhibited markedly greater CCT compared with fellow eyes. With regard to the clinical course of this thickening, Nishi et al. reported that optical correction resulted in a significant reduction in CCT in amblyopic eyes, concurrent with visual recovery [17]. These findings indicate that choroidal thickening is not a permanent structural abnormality but rather a plastic, reversible physiological adaptation to visual deprivation. The underlying mechanisms are likely multifactorial, involving biased emmetropization driven by interocular differences in visual input, compensatory responses of developing choroidal tissue, and dysregulation of neurovascular signaling [1]. Collectively, these results support the notion that choroidal thickening represents a structural phenotype intrinsic to treatment-naïve hyperopic anisometropic amblyopia.

A key finding of this study was that, despite the significant thickening of amblyopic eyes, the macular MBR and pulsatile component were significantly reduced. This observation indicates a “structure-function dissociation,” in which morphological thickening of the choroid does not correspond to enhanced functional perfusion and pulsatility. Although evidence regarding choroidal blood flow in hyperopic anisometropic amblyopia remains limited, previous studies—including a case report—demonstrated that macular MBR was reduced prior to treatment and increased following visual improvement [10]. Decreased BS generally indicates attenuated transmission of pulsatile energy to the vascular bed [9,18]. The concurrent reduction in macular MBR and BS observed in this cohort suggests that, despite structural thickening, choroidal circulation in amblyopic eyes, may exist in a “functionally inactive or suppressed state.”

In a previous study using LSFG, Itokawa et al. examined pediatric anisometropic amblyopia and reported no significant difference in choroidal blood flow between amblyopic and fellow eyes in the peripapillary region [15]. This discrepancy is likely attributable to differences in both measurement site and disease stage. Although their study assessed peripapillary choroidal blood flow, the present study focused on macular blood flow. Furthermore, their patients were in the recovery phase, having achieved a visual acuity of 20/20 or better following treatment. Meanwhile, the present cohort consisted of children in the active phase of amblyopia with residual visual impairment (20/30 or worse). This distinction is critical. Although blood flow demand may have already normalized in the recovered eyes reported by Itokawa et al., local macular hypoperfusion is more readily detectable in the present cohort, in which visual dysfunction remained evident.

This local hemodynamic abnormality is likely driven by two key factors. First, demand-driven downregulation of vascular function may occur due to reduced metabolic demand. The observed reduction in macular MBR and BS, despite significant choroidal thickening, suggests a dysfunction in neurovascular coupling. In amblyopic eyes, neuronal activity is diminished, leading to lower metabolic requirements [1,2], which likely contributes to reduced pulsatility via neurovascular coupling. This hypothesis is supported by observations of reduced retinal vessel density in hyperopic anisometropic amblyopic eyes compared with fellow eyes [19]. Second, relative choroidal hypoperfusion may result from structural remodeling. Previous studies have reported that choroidal vascular density decreases as the choroid thickens, specifically in amblyopic eyes [6]. Furthermore, Hui et al. recently demonstrated that, although both luminal and stromal areas were increased in amblyopic eyes, the choroidal vascularity index was lower compared with controls [20]. Collectively, these structural and functional findings suggest that, in pediatric hyperopic anisometropic amblyopia, the blood supply does not adequately match the expanded choroidal vascular bed, resulting in relative hypoperfusion or congestion.

With regard to the validity of the measurements, LSFG employs a diode laser with a wavelength exceeding 800 nm, allowing for penetrating deep ocular tissues [8]. Iwase et al. demonstrated a significant positive correlation between subfoveal choroidal thickness and MBR in 241 healthy eyes, indicating that physiologically thicker choroids are generally associated with “higher” blood flow [21]. If tissue thickness were the primary factor attenuating the LSFG signal, MBR would expected to decrease as thickness increases, which directly contradicts these findings. Therefore, the reduced blood flow despite choroidal thickening observed in this study is unlikely to reflect a measurement artifact and may instead indicate pathological neurovascular uncoupling.

This study has some limitations. First, its retrospective cross-sectional design precluded the evaluation of causal relationships or longitudinal changes in blood flow and CCT associated with treatment. Future prospective studies are needed to monitor the trajectory of macular MBR and CCT following therapeutic intervention for amblyopia. Second, the sample size was small, limiting the ability to perform subgroup analyses. Additionally, the broad age range of 2–10 years may introduce confounding effects due to developmental changes in axial length and choroidal thickness. Validation in a larger, more homogenous cohort is warranted. Third, the technical limitations of LSFG should be considered. LSFG does not allow for separate assessment of blood flow in the choriocapillaris and the medium or large vessel layers (Sattler’s and Haller’s layers). To elucidate which vascular layer undergoes structural remodeling and how this contributes to reduced blood flow, future studies should incorporate combined analyses using OCTA or en-face OCT. Furthermore, all measurements were conducted under pharmacological cycloplegia. While these agents can potentially influence choroidal hemodynamics, we employed an interocular comparison design where both the amblyopic and fellow eyes were examined under identical conditions. This approach minimizes the potential for pharmacological bias, although the exact differences compared to a non-mydriatic state remain a subject for future investigation.

## 5. Conclusions

This study demonstrated that eyes with hyperopic anisometropic amblyopia exhibit characteristic choroidal thickening, whereas macular choroidal blood flow and pulsatility are significantly reduced. This “structure-function dissociation” likely reflects reduced metabolic demand due to decreased neuronal activity and relative blood flow stagnation associated with structural remodeling. These findings suggest that the pathophysiology of amblyopia involves not only the underdevelopment of central neural circuits but also local hemodynamic dysregulation in the macula, providing a novel perspective on the disease. Future longitudinal investigations are warranted to determine how therapeutic interventions and visual recovery influence the normalization of this circulatory dissociation. Moreover, these hemodynamic parameters may serve as objective clinical biomarkers for monitoring the physiological status of amblyopic eyes during treatment. Future research incorporating larger, age-matched cohorts is necessary to clarify whether early normalization of macular blood flow can predict long-term visual outcomes and to overcome the inherent limitations of our cross-sectional study.

## Figures and Tables

**Table 1:** Baseline demographics and ocular characteristics of the study participants.

Parameter	Amblyopic Eyes (*n* = 18)	Fellow Eyes (*n* = 18)	*p*-Value
BCVA (logMAR)	0.42 ± 0.31	0.018 ± 0.18	<0.0001 ^‡^
Spherical equivalent (D)	+6.49 ± 1.26	+2.82 ± 2.30	<0.0001 ^†^
Axial length (mm)	20.74 ± 0.66	22.12 ± 1.15	<0.0001 ^†^
IOP (mmHg)	14.7 ± 3.0	15.3 ± 3.0	0.229
OPP (mmHg)	35.4 ± 11.9	34.8 ± 11.5	0.245
CRT (µm)	207.9 ± 19.8	203.1 ± 15.3	0.133

Values are expressed as the mean ± standard deviation. ^†^ *p*-value derived using the paired *t*-test. ^‡^ *p*-value derived using the Wilcoxon signed-rank test. Abbreviations: BCVA, best-corrected visual acuity; logMAR, logarithm of the minimal angle of resolution; D, diopters; IOP, intraocular pressure; OPP, ocular perfusion pressure; CRT, central retinal thickness.

## Data Availability

The data presented in this study are available on request from the corresponding author due to privacy.

## Abbreviations

The following abbreviations are used in this manuscript:

AU	Arbitrary unit
BCVA	Best-corrected visual acuity
BS	Beat strength
CCT	Central choroidal thickness
CRT	Central retinal thickness
D	Diopters
EDI-OCT	Enhanced depth imaging optical coherence tomography
IOP	Intraocular pressure
LSFG	Laser speckle flowgraphy
logMAR	Logarithm of the minimum angle of resolution
MAP	Mean arterial pressure
MBR	Mean blur rate
NCT	Non-contact tonometer
OPP	Ocular perfusion pressure
ROI	Region of interest
SBP	Systolic blood pressure
DBP	Diastolic blood pressure
SD-OCT	Spectral-domain optical coherence tomography
